# Improving the Trustworthiness of Interactive Visualization Tools for Healthcare Data through a Medical Fuzzy Expert System

**DOI:** 10.3390/diagnostics13101733

**Published:** 2023-05-13

**Authors:** Abdullah M. Albarrak

**Affiliations:** College of Computer and Information Sciences, Imam Mohammad Ibn Saud Islamic University, Riyadh 13318, Saudi Arabia; amsbarrak@imamu.edu.sa

**Keywords:** interactive visualization, healthcare data, data visualization, trustworthiness assessment, decision making

## Abstract

Successful healthcare companies and illness diagnostics require data visualization. Healthcare and medical data analysis are needed to use compound information. Professionals often gather, evaluate, and monitor medical data to gauge risk, performance capability, tiredness, and adaptation to a medical diagnosis. Medical diagnosis data come from EMRs, software systems, hospital administration systems, laboratories, IoT devices, and billing and coding software. Interactive diagnosis data visualization tools enable healthcare professionals to identify trends and interpret data analytics results. Selecting the most trustworthy interactive visualization tool or application is crucial for the reliability of medical diagnosis data. Thus, this study examined the trustworthiness of interactive visualization tools for healthcare data analytics and medical diagnosis. The present study uses a scientific approach for evaluating the trustworthiness of interactive visualization tools for healthcare and medical diagnosis data and provides a novel idea and path for future healthcare experts. Our goal in this research was to make an idealness assessment of the trustworthiness impact of interactive visualization models under fuzzy conditions by using a medical fuzzy expert system based on an analytical network process and technique for ordering preference by similarity to ideal solutions. To eliminate the ambiguities that arose due to the multiple opinions of these experts and to externalize and organize information about the selection context of the interactive visualization models, the study used the proposed hybrid decision model. According to the results achieved through trustworthiness assessments of different visualization tools, BoldBI was found to be the most prioritized and trustworthy visualization tool among other alternatives. The suggested study would aid healthcare and medical professionals in interactive data visualization in identifying, selecting, prioritizing, and evaluating useful and trustworthy visualization-related characteristics, thereby leading to more accurate medical diagnosis profiles.

## 1. Introduction

### 1.1. Overview

The fourth industrial revolution has completely rethought the way the medical industry has been organized in the past. A lot of the fight against the COVID-19 epidemic has been carried out with the help of Industry 4.0 and its more advanced information and communications technologies (ICTs) [1]. During this pandemic, information and communication technology have helped solve a number of problems and led to some promising solutions. Since the early days of CT scanners and MRIs, there has been a significant advancement in the field of medical diagnostics. The field of medical diagnostics has recently shifted its focus to interactive visualization. Emerging technologies, such as artificial intelligence and deep learning, are utilized, together with data sets from medical imaging, to assist in the creation of interactive visualization models. At the heart of the Industry 4.0 initiative is the idea of interactive visualization, which can be achieved with the help of different artificial intelligence (AI) and decision-making systems [2].

**Figure 1:**
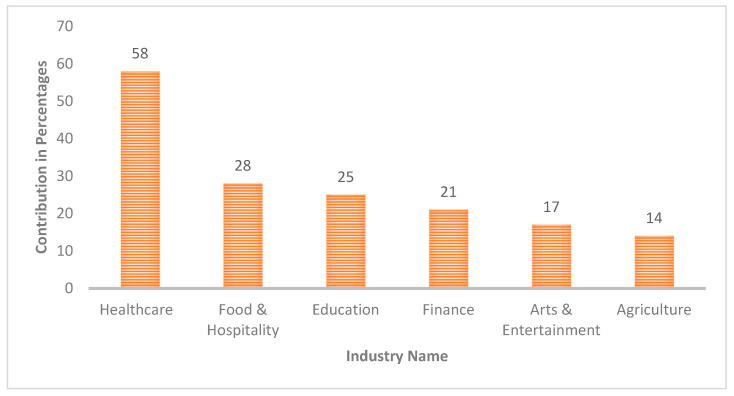
Statistics of data visualization markets in various industries.

AI is widely used as a result of the Industrial Revolution and technology’s significant advancements [3]. AI systems are widely used in a variety of industries, including healthcare, and have demonstrated tremendous effectiveness in the interpretation of complicated patterns [4]. Most people agree that AI systems are powerful tools in Industry 4.0, and they have been used a lot in the fight against this pandemic. The interactive visualization tools developed with the help of AI techniques have been used, for example, to analyze complicated medical diagnostics, lessen the effects of the epidemic, figure out the best treatment, and look for viruses by analyzing the symptoms of patients using medical imaging tools, such as CT scans and X-rays. Many examples of tasks where AI applications operate as well as or better than humans include the analysis of medical pictures and the correlation of symptoms and biomarkers from electronic medical records (EMRs) with the diagnosis and prognosis of the disease [5]. The real figures for the number of healthcare practitioners who are employing visualizations to clearly communicate data in medical diagnosis are presented in Figure 1.

According to Figure 1, about 58 percent of healthcare practitioners use interactive data visualizations for medical diagnosis and treatment of critical patients, which is more than in any other industry. Further, G. Stiglic et al. state that an AI system with a higher level of interpretability is one that end users will have an easier time understanding and in explaining future forecasts [6]. In addition, interpretable AI systems enable healthcare professionals to make decisions that are reasonable and driven by data, thereby providing personalized decisions that have the potential to ultimately lead to a higher quality of diagnosis service in the healthcare industry. The main goal of competitive health is to improve healthcare performance and eventually help people by providing them with good and speedy health services. This can be attained by pushing for higher standards of achievement, encouraging people to be active, and teaching people how to reach for good health services and deal with different services [1]. The information comes from patients as well as professionals working in the medical field, and their actions are at the heart of competitive health. These healthcare data used for medical diagnosis should be trustworthy to achieve a trustworthy visualization, which further becomes a deciding factor for multiple crucial medical treatments [7,8].

### 1.2. Background

Assessment of trustworthiness is moving away from mathematical methods and towards AI and decision-making methods right now because AI is good at extracting complex features without the help of humans [9]. There are many opportunities to start creative projects and scientific research in this area of research, which is growing all the time. For instance, predictive analysis carried out by AI may be utilized in the service of improving one’s health [10]. Interactive healthcare data visualization tools have the ability to improve public health practice by addressing questions that were previously unanswerable in medical diagnosis. For instance, at Massachusetts General Hospital, they are utilizing 3D visualization of medical imaging in order to study the anatomical makeup of their patients. This procedure not only boosts the productivity of radiologists and ultrasonographers but also contributes to the successful outcome of efforts to save the lives of patients and improve healthcare data visualization tools [11].

Alfredo Vellido, in his work, states the problem of interpretability and visualization in AI systems for medical diagnostic purposes. He argued that medical experts should be integrated into the design of medical data analysis for the sake of good interpretation [12]. The health sector depends heavily on healthcare data, and trustworthy interactive data visualization can help doctors diagnose critical diseases, treat them, and make treatments more effective [11]. Healthcare and medical diagnostic data are essential components of the health industry. In addition to this, we are able to build better plans for healthcare thanks to big data. Trust management is a vital component of any healthcare business, whether the focus is on the health of individuals or teams. These techniques are dependent on the presence of professional healthcare teams in order to compete against their rivals. The modern approach to coaching makes use of enormous healthcare data sets in order to design effective strategies for both individuals and teams.

Dhillon et al. examined in one of their works the conceptual integration of traditional statistics and AI, with a primary emphasis on the research undertaken in the field of health. They found that researchers in the field of medicine might look at using AI to supplement traditional statistical methods of decision making in order to obtain additional trustworthy validation metrics [13]. In the healthcare industry, analyzing the trustworthiness of performance helps doctors and other professionals reach their goals by pointing out actions that can help guide decision making, improve performance, and get them started on the path to security and excellence.

Guangyue Zhang et al. emphasize in their work that technologies such as interactive healthcare data visualization and AI, along with decision making, have the potential to reduce the amount of information that management must process in the continuous monitoring of trustworthy healthcare software [14]. The process of visually representing healthcare data is the first step towards making sense of it. Users are able to tell stories through the use of data visualization by arranging data into a form that is simpler to comprehend, highlighting patterns, and drawing attention to outliers. An interactive healthcare data visualization should be able to tell a story in addition to filtering out irrelevant information and presenting the information that is important. To make an interactive healthcare data visualization, you need to find a good balance between how the tool looks and how it works. As a result of this, healthcare data analysts use a wide range of tools, such as graphs, diagrams, and maps, to understand and show healthcare data as well as the connections between them. Most of the time, the right method and how it is set up are needed to make healthcare data understandable.

### 1.3. Scope and Contributions

In addition, the evaluation of the trustworthiness of the interactive visualization achieved through fuzzy decision making in healthcare for medical diagnosis purposes is an ongoing process that is required to be carried out on a regular basis by industry professionals in order to test the security, effectiveness, and accuracy of these products. As a result, the goal of this research endeavor on our part is to evaluate the trustworthiness of interactive visualization achieved by means of fuzzy decision making in the medical field and to carry out an idealness evaluation while taking into account the constraints imposed by multi-criteria decision-making (MCDM) methodologies, since this problem is divided into different criteria [15,16]. For the purpose of this evaluation, the identification and selection of the pertinent characteristics are determined by the opinions of the specialists, which are provided in the second section of this paper.

The goal of this study is to comprehend how interactive healthcare data visualizations using a hybrid medical expert fuzzy system algorithm can be used in the healthcare industry to diagnose patterns. To perform rank analysis for various hybrid medical expert system classification algorithms employing decision-making analysis algorithms, such as fuzzy analytic network processing using TOPSIS, five different aspects of healthcare data visualization are used in this analysis [17,18,19,20]. Five alternatives to interactive visualization applications (SAS Visual Analytics, Tableau, QlikView, Bold BI, and DOMO BI) have been applied to the healthcare data in TOPSIS as alternatives in order to enhance the study’s findings. Further, the author of this study determined the sensitivity of the results, compared the results of the proposed method with three other methods, and analyzed the statistical significance. The remainder of the research paper consists of an introduction to interactive visualization using a hybrid medical expert system, a description of the computational methodology adopted, the results, discussion, and a conclusion.

### 1.4. Organization of the Paper

This article’s structure is as follows: The first section of the paper analyzes many trends and statistics from previous years to give the reader a summary and a sense of the relevance of the topic. As background, previous practitioners’ relevant studies are examined, in which their trustworthy interactive visualization tools are presented. The paper’s second portion discusses trustworthiness and interactive visualization tools. These components were given a network-like structure and ranked by impact probability. The fuzzy ANP-TOPSIS approach was used to analyze the suggested network problem numerically. The final section presents this study. In the fifth section, a discussion with a comparative study is given. The detailed debate and study limitations are summarized in a conclusion.

## 2. Materials and Methods

### 2.1. Trustworthy Interactive Visualization for Healthcare Data

Interactive data visualization in the healthcare sector is dependent on advanced current technology, which enables professionals from a variety of professions to effectively take decisions in medical diagnosis [21,22,23,24]. Interactive visualization tools help healthcare practitioners comprehend trends that have occurred in the past as well as those that are occurring in the present, in addition to helping them predict and anticipate future trends and directions. Interactive healthcare data visualization, in its broadest sense, refers to the practice of displaying information and healthcare data for medical diagnosis purposes in a variety of formats, including graphs, charts, diagrams, and photographs [25,26,27,28]. These interactive healthcare data visualization approaches can make it simple for healthcare providers to recognize and comprehend patterns, trends, and outliers in healthcare data [2].

Trustworthiness issues in interactive visualization techniques have become increasingly relevant in many areas of healthcare, particularly with regard to assisting medical professionals in the formulation of vitally important clinical choices for the health of patients and communities. A healthcare organization is able to transform raw healthcare data into graphs and then exhibit them in charts by utilizing a variety of approaches for healthcare data visualization [14]. This enables the organization to perform rapid analysis of trends and patterns.

A trustworthy tool or piece of software provides efficient and interactive visualization by utilizing systems associated with healthcare, examining threats, and responding to incidents swiftly and instinctively. To solve the many challenges that come with healthcare data visualization, specialists and researchers have brought a variety of assurance issues to light [17]. The overall level of trustworthiness of healthcare interactive visualization tools can be broken down into their component parts. The combination of these characteristics can vary depending on who is receiving treatment. For consumers, for instance, trustworthiness in healthcare visualization tools consists primarily of a perceived amount of control and privacy. On the other hand, for healthcare professionals, trustworthiness in visualization involves a far bigger and more diverse collection of concerns, such as reliability and a transparent healthcare data storage policy. When compared to the sets of elements that affect trustworthiness in the general healthcare data visualization domain, the sets of factors that affect trustworthiness in a healthcare portal are unique. In order to ensure that interventions are trustworthy, it is necessary to carry out independent research on the topic of trustworthiness in healthcare interactive visualization tools as a separate topic.

A case study was conducted on a few different alternatives in order to enhance the performance of healthcare interactive visualization tools and rank the characteristics of healthcare interactive visualization tools in descending order of importance [8]. A consensus was reached among the authors regarding the characteristics to be used in the evaluation of healthcare interactive visualization tools, and these conclusions informed the decision-making process for identifying and selecting the criteria. For the purpose of this work, five characteristics of trustworthiness, four characteristics of visualization design, and three characteristics of interactive widgets were taken into consideration for the idealness assessment. The decision that experts in a particular field make collectively is what drives the process of alternative selection. Further, alternatives were chosen as per the popularity of interactive visualization tools among healthcare professionals. The authors of the study used fuzzy logic for this evaluation, so each healthcare data visualization was assigned a value between 0 and 1 for each characteristic.

**Figure 2:**
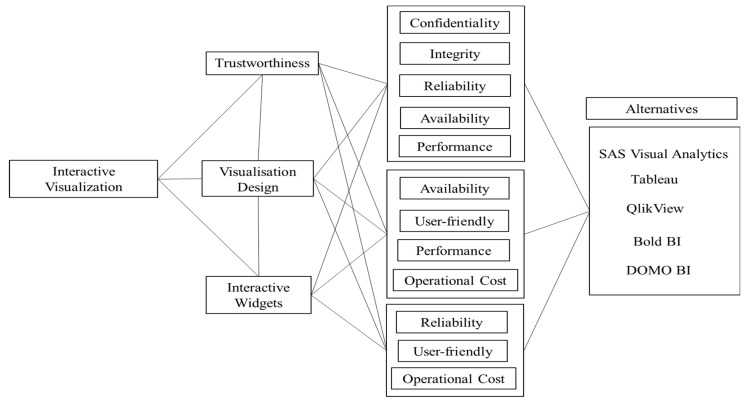
Assessment characteristics for interactive visualization of healthcare data.

Moreover, with regard to our specified characteristic set, each healthcare data visualization tool gained a value between 0 and 1 for each characteristic. In addition, the results of the evaluators’ subjective cognition in linguistic words for each healthcare data visualization characteristic were based on the scale and the opinions of the experts, both of which are explained in the methodology portion of the paper. This work presents, on the basis of the identified characteristic set, the process of evaluating the six tools used for healthcare as well as the quantitative outcomes of that evaluation. The characteristics that were found and the alternatives are shown in Figure 2. The figure that follows discusses both the subsection description and the significance of the traits that were identified.

Characteristics at the first level are further divided into sub-characteristics. Trustworthiness is affected by confidentiality, integrity, reliability, availability, and performance [19]. Visualization design is affected by availability, user friendliness, performance, and operational cost. Interactive widgets in a visualization tool depend on its characteristics, such as reliability, user friendliness, and operational cost. The first-level characteristics depend on each other for the best interactive visualization tool building. Hence, after creating its dependencies, it becomes a network-like structure problem. For such a multiple-criteria network-like structure problem, the Analytic Network Process (ANP) is a mathematical theory that was developed by Saaty [28] that is helpful for forecasting and presenting the influence of multiple decision criteria, their interactions, and their relative weights. ANP was named after the nature of its problem, which is a network-like structure of different criteria. To calculate the different weights of characteristics associated with this problem, we used ANP with fuzzy logic and TOPSIS for ranking the alternatives.

#### 2.1.1. Trustworthiness

Interactive visualization tools are having an increasingly substantial impact on clinical decisions and diagnoses as a result of the significant increase in the number of healthcare solutions. On the other hand, there is scant evidence to support the notion that software can be trusted. An application or tool is said to have trustworthiness when it possesses the qualities that make it trustworthy for others, such as healthcare professionals. There are different characteristics of trustworthiness that contribute to building a trustworthy visualization tool. We identified five important characteristics, the details of which are as follows:

Confidentiality: To maintain healthcare data privacy, it must be protected from digital and physical intrusion. Confidentiality is closely tied to other aspects of information privacy, such as who can see, share, and use specific pieces of healthcare data. Information with a low level of confidentiality may be considered “public” or innocuous if disclosed to a larger audience. High-confidentiality information must be protected against disclosure for the sake of avoiding identity theft, account and system compromise, legal or reputational damage, and other undesirable outcomes. The importance of privacy in keeping healthcare data visualization trustworthy is thus demonstrated.

Integrity: Healthcare data integrity is vital because so much depends on it. A dataset error in healthcare data can impact a clinician’s decision making. Healthcare data integrity means accuracy, completeness, and consistency. Healthcare data integrity includes security for regulatory compliance. It decreases consumer assurance and trustworthiness. There are several data integrity threats. Copy-transferred data should not be modified between updates. Error-checking and validation maintain data integrity when sent or reproduced without alteration.

Reliability: For a clinician or healthcare expert to use a visualization tool to ensure the accuracy of healthcare data, the data must be complete and correct. This is what is meant by “data reliability.” One of the main goals of data integrity programs, which are also used to maintain data security, data quality, and regulatory compliance, is to make sure data is trustworthy. The term “data reliability” refers to how consistent information is between different databases, apps, or platforms. It also has to do with how trustworthy the source of information is. If the data are trustworthy enough, a trustworthy figure will always be right. Thus, it can be seen as a sign of honesty.

Availability: The frequency with which healthcare data can be used by a healthcare provider organization or a partner is a measure of data availability. A healthcare provider organization runs more smoothly when clinicians have access to data at all times. Important aspects of data availability include access to the data and a steady stream of data. Data that cannot be accessed are as good as useless. As a result, data availability is the only factor that can influence the trustworthiness of interactive data visualization. If workers were to have problems obtaining firm data, productivity would take a hit. Data accessibility is critical for most modern enterprises. The good news is that, by following data availability best practices, a forward-thinking firm may enjoy all the advantages of having sufficient data availability.

#### 2.1.2. Visualization Design

User-Friendly: The goal of good visualization tool design is to ensure that a product is not only functional but also pleasurable and simple to use for healthcare experts. The interactive visualizations could be understood by every level of health expert, be it a researcher in healthcare or a doctor. The goal is to guarantee that every consumer of an interactive visualization tool is completely content with their experience using the product. An improved user experience is the result of a design that facilitates the user’s goals and duties. The design must be simplified, the instructions must be clear and succinct, and the learning curve must be minimized.

Operational Cost: Visualization tool developers have never been in a position where they are not under pressure to cut expenses and maximize investment. This pressure has only risen as a result of COVID-19, as more and more hospitals and healthcare organizations have sped up their digitalization efforts in an effort to preserve a viable, virtual corporate presence. These operational cost optimizations have a direct impact on the design, trustworthiness, and interactive widgets of tools. Hence, operational cost is an important criterion for measuring a trustworthy and interactive visualization tool for healthcare professionals.

#### 2.1.3. Interactive Widgets

The goal of interactive visualization is to improve the way in which people engage with information by using graphical representations of healthcare data. The term “interactive visuals” can also apply to the graphical displays that are employed by various technologies for analytics and business intelligence. The majority of the time, these representations are implemented in the form of interactive widgets. These widgets offer a simple method for comprehending insights that may be based on data that are constantly shifting. In order for healthcare data visualizations to be called interactive, they need to incorporate some sort of human input (such as the ability to click on a button or move a slider), and their response times need to be fast enough to demonstrate a genuine connection between the healthcare data input and the visual output.

Performance: Problems with performance are characterized by a reaction time for output that is significantly longer than the time that is anticipated for its execution in a healthcare interactive visualization tool. The performance could be caused by untrustworthy third-party healthcare data, such as databases or hardware, or it could be caused by the design of the visualization. Hence, performance issues can be affected by trustworthiness, visualization design, and interactive widgets as well.

All of the features that were covered earlier have some bearing on interactive visualization tools. In addition, each of the mentioned traits, by virtue of the implicit specifications that they carry, plays an important part in the ideality of interactive visualization for healthcare as a whole. The authors of the study began by compiling a list of the multiple characteristics that were pertinent to the investigation. Following that, a conversation was had with the team of healthcare and security tool development experts about finalizing the characteristics set. After having a group discussion about all of the detected features, the specialists deleted any characteristics that were deemed unnecessary or inconsistent. The individual disagreements that arose among the experts over the characteristic selection were brought to a minimum, and at the end of this expert group debate, a collection of trustworthy healthcare data visualization characteristics was decided upon. So, each of these characteristics was taken into consideration for this analysis.

### 2.2. Methodology

The Analytic Network Process (ANP) of MCDM can solve networked decision-making problems. T.L. Saaty invented the technique in 1965. The approach has changed and improved since then [28]. It estimates the relative relevance of criteria (characteristics). It helps specialists choose the judgement that best fits their goal and understanding of the situation. Fuzzy data improve this process and helps elicit more accurate healthcare data [29].

Fuzzy sets are crucial here. Fuzzy sets accurately reflect decision makers’ foggy preferences. Fuzzy logic can eliminate doubts when defining an element’s membership in a fixed set is challenging. This is very useful for categorizing elements. Fuzzy logic rarely addresses such issues [30]. TOPSIS also produces the highest alternate rating [8]. Its main draw is this ability. Thus, integrating fuzzy logic with the ANP-TOPSIS methodology makes this study more effective and allows it to be used to evaluate the efficacy of interactive visualization achieved through the software of a hybrid medical expert system.

**Figure 3:**
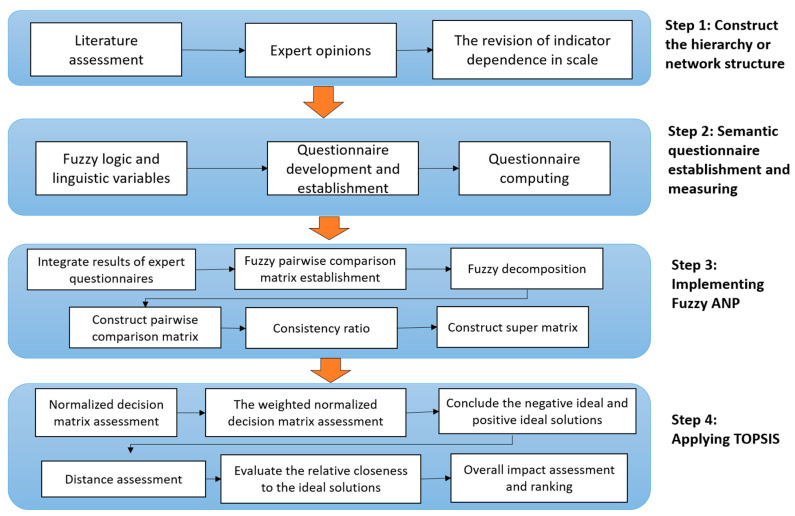
Flowchart of the proposed methodology.

This article evaluates each visualization tool’s success using the ANP-TOPSIS main methods. The fuzzy TOPSIS approach first determines the weight of each characteristic for the fuzzy ANP method. To establish correct weights, correlations between attributes are examined. After determining these attributes’ key weights, the fuzzy TOPSIS technique is used to assess decision makers’ social and economic performance and risk. These methods require interactive visualization specialists and a hybrid medical expert system. The specialists must have at least three years of experience selecting, managing, and conceptualizing interactive visualizations utilizing a hybrid medical expert system. Figure 3 shows the integrated fuzzy ANP TOPSIS method:

#### 2.2.1. Fuzzy Analytic Network Process

There is a significant potential for fuzzy-based ANP-TOPSIS to tackle MCDM challenges generated by imprecise and uncertain healthcare data [28,30]. When applied in a fuzzy environment, ANP yields characteristic weights that are more accurate, which in turn leads to outcomes that are more beneficial [28]. The TOPSIS method, when applied to problems involving MCDM, is one of the better-known techniques for ranking available solutions [28]. In this particular study, a total of 5 interactive visualization tools were used as alternatives and 12 characteristics of interactive visualization were used as criteria. When identifying and selecting the qualities, both the views of the specialists and well-known research works were taken into consideration.

Thomas Saaty [28] came up with the idea of incorporating the ANP, which is an extension of the AHP (Analytic Hierarchy Process). It enhances the capacity to handle interactions and dependencies between characteristics and sub-characteristics, which might alter the weights that are allocated to them. This can be a challenge when trying to build a model. Despite the many attempts to alter it so that it can account for erroneous human judgments, the ANP is severely constrained when it comes to evidentiary assessment fractions. The fundamental concept that underpins this is that the aggregation strategy utilized by the ANP is a reasonably straightforward one that can be applied to intervals as well as locally fuzzy priorities. However, in order to carry out the supermatrix priority derivation technique in the ANP, one must be familiar with complex real-number matrix operations. Nevertheless, none of the currently available methods for determining interval or fuzzy local priorities give results that can be incorporated into the calculation of the ANP matrix. This is the case despite the fact that these methods have been developed. In order to solve the unpredictability that surrounded the preferences of experts in a pairwise comparison matrix, the fuzzy ANP (PWCM) was developed. The following is a rundown of the various stages of the fuzzy ANP strategy that will be used in the calculation of the weights of the qualities:

Step 1: The process of constructing the network of the problem involves characterizing the relationships that exist among its numerous parts.

Step 2: In the following step of the procedure, triangular fuzzy numbers (TFNs) based on the scale proposed by Saaty [28] are used to compare various linked properties of the network pairwise and create comparison matrixes. The linguistic term “comparison” is used in this study, and the appropriate TFNs were assigned [30].

Step 3: Creating the Supermatrix.

In order to construct the supermatrix, it is necessary to ascertain the relative importance of each characteristic and sub-characteristic. As each comparison matrix’s characteristics have a triangular fuzzy structure, the respective weights are calculated using Lotfi Zadeh’s proposed extent analysis method [28]. This method consists of the following steps and is necessary because of the triangular, fuzzy membership structure.

Step 3.1: Finding the value of the fuzzy artificial degree for each of the following qualities is the first step in Zadeh’s technique:

Let us assume that *Pw* is a pairwise comparison matrix in (Equations (1)–(5)):

$$Pw={\left[Q_{ij}\right]}_{r\times s},Q_{ij}=\left(l_{ij},m_{ij},u_{ij}\right)$$

(1) i=1,2…..r j=1,…..s

(2) $$S_{i}=\sum_{j=1}^{s}Q_{ij}\times {\left[\sum_{i=1}^{r}\sum_{j=1}^{s}Q_{ij}\right]}^{-1}$$

(3) $$\mathrm{where}\;\sum_{j=1}^{s}Q_{ij}=\left(\sum_{j=1}^{s}l_{j},\sum_{j=1}^{s}r_{j},\sum_{j=1}^{s}u_{j}\right)\forall i,$$

(4) $$\sum_{i=1}^{r}\sum_{j=1}^{s}Q_{ij}=\left(\sum_{i=1}^{r}\sum_{j=1}^{s}l_{ij},\sum_{i=1}^{r}\sum_{j=1}^{s}m_{ij},\sum_{i=1}^{r}\sum_{j=1}^{s}u_{ij}\right),\;\mathrm{and}$$

(5) $${\left[\sum_{i=1}^{r}\sum_{j=1}^{s}Q_{ij}\right]}^{-1}=\left(\frac{1}{\sum_{i=1}^{r}\sum_{j=1}^{s}u_{ij}},\frac{1}{\sum_{i=1}^{r}\sum_{j=1}^{s}m_{ij}},\frac{1}{\sum_{i=1}^{r}\sum_{j=1}^{s}l_{ij}}\right)$$

Step 3.2: Calculating the relative likelihood of each Si over other characteristics (Equation (6)).

(6) $$V\left(S_{1}\geq S_{2}\right)=\left\{\begin{array}{c} 1\;\;\;if\;m_{1}\geq m_{2}\\ 0\;\;\;if\;l_{1}\geq u_{2}\\ \frac{l_{2}-u_{1}}{\left(m_{1}-u_{1}\right)-\left(m_{2}-l_{2}\right)}\;otherwise \end{array}\right.$$

Step 3.3: When using Equations (7) and (8), it can be difficult to determine how much weight each characteristic ought to be given in terms of the probability that a convex fuzzy number is greater than k other convex fuzzy numbers.

(7) $$W_{i}^{\prime }=V\left(S_{k}\geq S_{1},S_{2},\ldots \ldots S_{r}\right)=\underset{i=1,2,\ldots .k,..r}{\mathrm{Min}}\left(S_{k}\geq S_{i}\right)$$

(8) $$w^{\prime }=(W_{1}^{\prime },W_{2}^{\prime },\ldots ..,W_{r}^{\prime })$$

Step 3.4: Estimating the normalized weight vector using (Equation (9)).

(9) $$w=(W_{1},W_{2},\ldots ..W_{r})$$

Once the weights of each pairwise comparison matrix have been computed, the supermatrix can be created according to Equation (10):

(10) $$W^{\prime }=\begin{array}{c} 1=Goal\\ 2=Criteria\\ 3=Sub-criteria \end{array}\left[\begin{array}{ccc} 0 & 0 & 0\\ W_{21} & W_{22} & 0\\ 0 & W_{32} & W_{33} \end{array}\right]$$

Step 4: Computing the actual weight vector of each sub-characteristic (Equation (11)).

(11) $$W=\underset{x\to \infty }{\lim}W^{\prime 2k+1}$$

#### 2.2.2. Fuzzy Technique for Order of Preference by Similarity to Ideal Solutions

The fuzzy TOPSIS method was first put forth by M. M. D. Widianta et al. [31], and since then it has been widely used for evaluating alternatives in a range of contexts. This technique can be used to rank choices according to how similar or close they are to the ideal response. On this page [29], there is a lot of information about how to use fuzzy TOPSIS.

Step 1: Calculating the decision matrix that has been normalized.

Supposing that *D_nd_* is the normalized fuzzy decision matrix (Equation (12)):

(12) $$D_{nd}={\left[d_{ij}\right]}_{r\times s}\;i=1,\ldots \ldots .r\;j=1,\ldots \ldots ,s$$

Every aspect of decision making, with the exception of the decision-making process itself, has been standardized according to the category that best fits each principle. The principle decides whether it is a benefit principle or a cost principle, which means that an increase in magnitude is advantageous in the first category; however, in the second category, a decrease in size is advantageous. To calculate each one, the following equations are used, which are classified by each component of the normalized decision-making process (Equation (13)):

(13) $$d_{ij}=\left(\frac{l_{ij}}{u_{j}^{+}},\frac{m_{ij}}{u_{j}^{+}},\frac{u_{ij}}{u_{j}^{+}}\right)$$

where $u_{j}^{+}$ is the maximum $u_{ij}$ for the benefit characteristic (Equation (14)).

(14) $$d_{ij}=\left(\frac{l_{j}^{-}}{u_{ij}},\frac{l_{j}^{-}}{m_{ij}},\frac{l_{j}^{-}}{l_{ij}}\right)$$

where$,\;l_{j}^{-}$ is the minimum $l_{ij}$ for the cost characteristic.

Step 2: Calculating the weighted normalized decision matrix.

The weight of the characteristic is represented as wi, and the weighted normalized decision matrix is determined as follows (Equation (15)):

(15) $$V={\left(v_{ij}\right)}_{r\times s}\;\mathrm{where}\;v_{ij}=r_{ij}\times w_{ij}\forall j=1,\ldots \ldots ,s,and\;i=1,\ldots ..,r$$

Step 3: The fuzzy positive ideal and the negative ideal as potential answers are suggested in this step.

$FPI=\left(v_{1j}^{+},\ldots ..v_{ij}^{+}\right)$ for the benefit characteristic; $FPI=\left(v_{1j}^{-},\ldots ..v_{ij}^{-}\right)$ for the cost characteristic.

Where $v_{i}^{+}$ is the maximum $v_{ij}^{-}$, $v_{ij}^{-}$ is the minimum $v_{ij}^{+}$, and *i* = 1,….., *r*; *j* = 1,……, *s*.

Step 4: Calculating the distance of each alternative from positive ideal and negative ideal solutions (Equations (16) and (17)).

(16) $${dia}_{j}^{+}=\sum_{j=1}^{n}{dia}_{v}\left(v_{ij},v_{j}^{+}\right)i=1,\ldots \ldots ,r$$

(17) $${dia}_{j}^{-}=\sum_{j=1}^{n}{dia}_{v}\left(v_{ij},v_{j}^{-}\right)i=1,\ldots \ldots ,r$$

The distance between two TFNs can be calculated (Equation (18)):

(18) $$dia\left(\tilde{A},\tilde{B}\right)=\sqrt{\frac{1}{3}\left({\left(l_{A}-l_{B}\right)}^{2}+{\left(m_{A}-m_{B}\right)}^{2}+{\left(u_{A}-u_{B}\right)}^{2}\right)}$$

Step 5: This step is performed to calculate the closeness coefficient factor (CCF) (Equation (19)).

(19) $${CCF}_{j}=\frac{{dia}_{j}^{-}}{\left({dia}_{j}^{+}+{dia}_{j}^{-}\right)}$$

Step 6: Prioritizing the alternatives.

The algorithm that is used to determine the closeness coefficient gives more weight to the choice that has the highest *CCFj* value; hence, the one that has the highest *CCFj* value is the one that comes out on top in the ranking list.

## 3. Results

Interactive visualization tools provide users with a number of alternatives for visualizing healthcare data for the purpose of medical diagnosis that go beyond the traditional options of pie, bar, and line charts. Some examples of these possibilities are 3D medical imaging and micro-CT, as well as X-rays, scatter plots, and other types of visualizations developed for particular diagnosis. Graphical representations of healthcare data are made available to healthcare experts with these tools, enabling them to conduct medical diagnoses by interacting with the representations. In the following, we shall examine the significance of a hybrid medical expert system in interactive visualization by utilizing procedures that consist of fuzzy networks. The numerical analysis of this attempt will provide a quantitative evaluation of the results it generates.

As a direct result of this, the purpose of this work is to conduct a case study on five alternatives of interactive visualization tools in order to investigate the qualities that characterize their appropriateness from the point of view of interactive visualization. The approach that was selected for this inquiry incorporates, as component elements, all of the possible identifiers that can be given, in addition to rating evaluations for each one. This was decided upon before the investigation began. In addition, these five interactive visualization tools were selected as alternatives for their comparative trustworthiness evaluation based on the decision that was arrived at by reaching a consensus amongst the owners of the relevant domains and the experts in these fields. This was done to ensure that the best possible results would be obtained from the evaluation. In order to make this study more productive and corroborative, an ANP-TOPSIS analysis was conducted under fuzzy settings. Under fuzzy settings, an evaluation of the ideality of a hybrid medical expert system was carried out with the support of ANP-TOPSIS. The evaluation focused on the context of interactive visualization. Following the equations indicated in the technique section (Equations (1)–(19)), the method was used to carry out this evaluation.

Following the conversion of the linguistic phrases to quantitative values (Steps 1–4 and Equations (1)–(11)), the values were then further refined into fuzzy-based, crisp numerical values (Step 5). Following this, numerical calculations were carried out in order to construct a pairwise comparison matrix, and the outcomes of these calculations, a summary of which can be found in Table 1, are shown below. The algorithm proceeded through the process of implementing fuzzy integer values and then subsequently transitioned into fuzzy-based crisp numeric values so that the final results could be written down in Table 1. Following this, numerical calculations were carried out in order to build a pairwise comparison matrix, and the results of these calculations, which are summarized in Table 1 and are shown below, may be found in the next paragraph.

In order to obtain the final results displayed in Table 1, the methodology was modified to include fuzzy wrappers (Equations (1)–(5)), estimation of triangular numbers, and degree of possibility. At long last, the specialists arrived at the pairwise comparison matrix by using Equations (7) and (8). Table 1 displays the defuzzified values of the group’s characteristics. These values were computed using Equation (9), and the table was created. Table 2 displays the normalized weights of the group’s characteristics after calculating the local priority vectors, the weighted supermatrix, and the supermatrix formation. The complete findings of this inquiry are summarized below for convenience. After this, certain numerical calculations were carried out with the intention of calculating the absolute weight vector for row values and the traits that are the most important, as demonstrated in Equations (10) and (11). When the fuzzy data from the judgment matrixes were put together, a pairwise contribution matrix was the result. Further, Table 2 shows the combined results through the network.

The following part of the work provides a realistic assessment of the findings that were evaluated on particularly delicate interactive visualization software. The application of an ANP strategy while the conditions were fuzzy was used to obtain the combined weights of features; then, the software of TOPSIS, while the conditions were fuzzy, was used to obtain the global ranking of competing alternatives; this was carried out after the combined weights of features had been obtained. After this, we took the inputs on the technological data of five interactive visualization software systems and produced the summarized findings that are shown in Table 3 by including the standard scale that was defined in the technique. Passing the characteristic weights determined with the help of ANP to the TOPSIS technique when it is functioning in a fuzzy environment allows for the determination of the ranking order for the many options that can be chosen.

Following the completion of a number of procedures that served as intermediaries, the normalized fuzzy decision matrix for five alternatives of interactive visualization tools (SAS Visual Analytics, Tableau, QlikView, Bold BI, and DOMO BI) was discovered. The findings of the analysis are contained within this matrix. Equations (12)–(15) can be used to help with the calculation of the normalized performance values of the fuzzy decision matrix. Table 4 displays the definitive findings, which were computed by combining Equations (16) and (17) to establish the positive and negative idealness of each alternative with reference to each characteristic (Table 5 and Table 6). These equations were combined in order to ascertain the idealness of each alternative. The findings are arranged here according to the chronological order in which they were discovered. Equations (18) and (19) were used to compute the relative closeness score for each choice, which was then used to determine the degree of satisfaction; the results of this computation can also be found in Table 7. This was an after-thought calculation that was performed.

**Figure 4:**
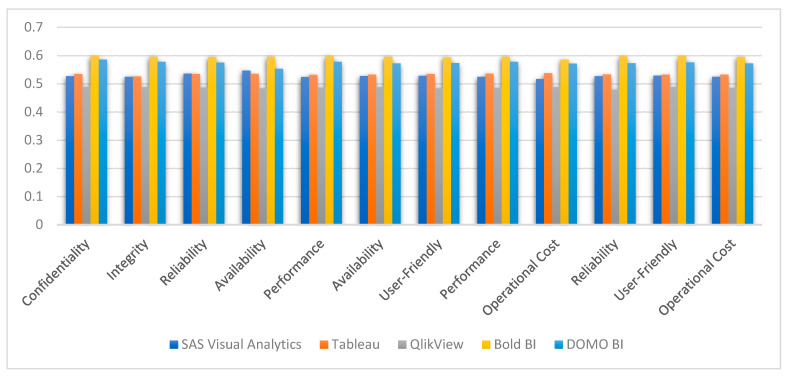
Sensitivity analysis in rankings when characteristics are varied from five percent.

The study was carried out on five alternatives of interactive visualization tools, and the results showed that classification is more ideal and effective when it comes to dealing with interactive visualization. The evaluation was carried out on the basis of the criteria that were chosen. It is of the utmost importance to determine whether or not the results achieved are stable when one is attempting to demonstrate a framework for making decisions based on a number of different criteria. This can be achieved by determining whether or not the framework that is being demonstrated is stable. The sequence in which alternatives are presented is heavily influenced by the weights that are given to the various selection criteria. Alterations to the proportional weights of the various selection criteria have the potential to cause fluctuations in the ranks if they are not carried out carefully. In order to evaluate the dependability of the findings, the authors conducted a sensitivity analysis by following the technique provided in [5]. This allowed the authors to validate their findings. The sensitivity of the interactive visualization software to variations in performance can be investigated by incrementally adding a 5% penalty to the weights of each selection criterion while they are being implemented one at a time. The results of the sensitivity analysis are shown in graphical form in Figure 4, which are presented here for convenience. The outcomes unequivocally demonstrate that they maintain their constancy.

## 4. Discussion

Our study proposed a novel approach to analyzing the trustworthiness of interactive visualization tools to assist healthcare experts in helping patients. After analyzing different tools for interactive visualization, we found the BoldBI visualization tool to fulfil the requirements of a trustworthy tool for patients as well as healthcare experts. To validate the results of this assessment, a comparative analysis was also performed with different methods of MCDM.

The use of a wide range of different methods of analysis can provide a conclusive means of determining whether or not the analyzed result and the projected method are superior. The author of this study has compared the results of the fuzzy ANP-COmplex PRoportional ASsessment (COPRAS) (Method 1), fuzzy ANP-VIekriterijumsko KOmpromisno Rangiranje (VIKOR) (Method 2), and fuzzy ANP-Elimination Et Choix Traduisant la Realité (ELECTRE) (Method 3) methodologies [2,6,9] with the results of the fuzzy ANP-TOPSIS technique to gauge the effectiveness of the proposed methodology. The results acquired through approaches such as fuzzy ANP-COPRAS, fuzzy ANP-VIKOR, and fuzzy ANP-ELECTRE are comparable to the results obtained through techniques such as fuzzy ANP-TOPSIS. The comparison is shown in a tabular representation in Table 8.

It is clear, based on the information presented in Table 8, that the outcomes acquired by employing fuzzy ANP TOPSIS are more useful than the outcomes gained by employing any of the other methodologies. Therefore, using the hybrid method of fuzzy ANP-TOPSIS is more effective than using the other techniques to solve this particular problem. The trustworthiness of interactive visualization models was investigated and characterized by a number of different research projects. There has not been any quantitative research carried out on the impact of interactive visualization models’ trustworthiness when used in fuzzy situations with a medical fuzzy expert system. 

In addition, Spearman’s correlation was tested using Equation (20) in order to determine whether or not there is a connection between the rankings that were produced utilizing the various approaches.

(20) $$R=1-\frac{6\sum d_{i}^{2}}{n\left(n^{2}-1\right)}$$

Here *R* denotes the Spearman’s rank correlation coefficient, 𝑑 is the difference between two ranks, and 𝑛 denotes the total number of observations considered. The theoretical values of Spearman’s ranking correlation coefficient run from +1 to 1. In this context, the sign indicates the nature of the link, whether it is positive or negative, and the value indicates the degree to which the association exists, as either strong, medium, or weak. The estimated values of the rank correlation coefficients calculated using Spearman’s method, symbolized by the letter R, were 0.9736, 0.9845, and 0.9736 for Method 1, Method 2, and Method 3, respectively. It is possible to draw the conclusion that there is a significant positive link between the rankings produced by the two techniques (the suggested approach with Method 1, Method 2, and Method 3), since the value is extremely close to +1.

The visualization of medical data is no longer an option in the field of healthcare; rather, it is required for all contemporary medical institutions. It is anticipated that over the next six years, the global market for healthcare data analytics will expand by a factor of 3.5, growing from USD 11.5 billion in 2019 to USD 40.8 billion in 2025 [32]. The number of healthcare data that exist on the planet is staggering, and it will only continue to increase in the future. Not only has the collection and analysis of large numbers of healthcare data become crucial to diverse groups of experts working in fields such as economics, space research, and climate change, it has also become crucial to individuals and communities. This is because of the importance of the information that can be gleaned from healthcare data. Imaging as a visualization widget in medicine has the potential to identify and diagnose a wide variety of ailments, from cancer to heart issues, which has the potential to save the lives of millions of people. MRIs and CT scans are also quite effective, but the 2D images that are produced by these procedures require the examining physician to make certain assumptions in order to properly diagnose the patient’s condition. Such types of visualization technology enable clinicians to build 3D images of MRI scans, which deliver crisper and higher-resolution photographs of blood vessels, organ tissues, and bones without the need for surgery. This results in significant savings in terms of both time and money.

## 5. Conclusions

Professionals and researchers have applied a wide range of techniques and strategies in order to build trustworthy and effective interactive visualizations. Their goal is to achieve this through the use of a variety of methods. A hybrid medical expert system is one of the best known of these technologies, and it plays an essential role. In this study, we investigated the trustworthiness of interactive visualization tools using the fuzzy ANP-TOPSIS method and ranked them accordingly. With the support of this methodology, researchers and developers will be able to design visualization tools that are more trustworthy. In spite of the results that we have obtained in this research work, there are other ways of making decisions that are based on a number of different methods (AHP, VIKOR, two-way fuzzy sets, etc.), and these other ways can be employed in order to achieve outcomes that are more productive. Further, the results of the empirical research that we conducted indicate that we have selected a method that is trustworthy for the purpose of this evaluation.

## Figures and Tables

**Table 1:** Pairwise comparisons matrixes of the groups.

Characteristic A/Characteristic B	Fuzzy Pairwise Comparisons Matrixes	Defuzzified Pairwise Comparisons Matrixes
Trustworthiness/Visualization Design	0.190, 0.170, 0.167	0.268
Trustworthiness/Interactive Widgets	0.364, 0.426, 0.44	0.277
Visualization Design/Interactive Widgets	0.319, 0.392, 0.401	0.370
Confidentiality/Integrity	0.148, 0.124, 0.118	0.015
Confidentiality/Reliability	0.348, 0.211, 0.106	0.390
Confidentiality/Availability	0.148, 0.113, 0.108	0.250
Confidentiality/Performance	0.192, 0.227, 0.222	0.390
Integrity/Reliability	0.152, 0.112, 0.108	0.350
Integrity/Availability	0.113, 0.075, 0.071	0.380
Integrity/Performance	0.226, 0.250, 0.253	0.450
Reliability/Availability	0.155, 0.122, 0.117	0.112
Reliability/Performance	0.192, 0.199, 0.196	0.248
Performance/Availability	0.306, 0.358, 0.373	0.390
Availability/User-Friendly	0.192, 0.199, 0.196	0.250
Availability/Performance	0.226, 0.250, 0.253	0.390
Availability/Operational Cost	0.155, 0.129, 0.124	0.233
User-Friendly/Performance	0.152, 0.112, 0.108	0.250
User-Friendly/Operational Cost	0.155, 0.093, 0.090	0.390
Performance/Operational Cost	0.113, 0.075, 0.071	0.350
Reliability/User-Friendly	0.192, 0.206, 0.203	0.380
Reliability/Operational Cost	0.148, 0.113, 0.108	0.450
User-Friendly/Operational Cost	0.192, 0.227, 0.222	0.112

**Table 2:** Weights of Interactive Visualization.

Characteristic	Symbols	Independent Weight of the Groups	Overall Weights through Network	Percentage	Priority
Characteristics of Group 1 at Level 1					
Trustworthiness	C1	0.259	0.259	25.90%	3
Visualization Design	C2	0.416	0.416	41.60%	1
Interactive Widgets	C3	0.326	0.326	32.60%	2
Characteristics of Groups 1, 2, and 3 at Level 2					
Confidentiality	C11	0.115	0.030	2.977%	12
Integrity	C12	0.363	0.094	9.398%	4
Reliability	C13	0.255	0.066	6.602%	10
Availability	C14	0.268	0.069	6.939%	9
Performance	C15	0.277	0.072	7.172%	8
Availability	C21	0.156	0.065	6.482%	11
User-Friendly	C22	0.267	0.111	11.094%	2
Performance	C23	0.265	0.110	11.011%	3
Operational Cost	C24	0.212	0.088	8.809%	5
Reliability	C31	0.248	0.081	8.075%	6
User-Friendly	C32	0.341	0.111	11.103%	1
Operational Cost	C33	0.233	0.076	7.586%	7

**Table 3:** Normalized decision matrix for alternatives with respect to criteria.

	SAS Visual Analytics	Tableau	QlikView	Bold BI	DOMO BI
Confidentiality	0.6578, 0.7570, 0.9190	0.6570, 0.7650, 0.9050	0.4560, 0.5330, 0.7330	0.8500, 0.9170, 0.9680	0.7720, 0.8560, 0.9450
Integrity	0.6490, 0.7640, 0.8800	0.6570, 0.7650, 0.9050	0.6578, 0.7570, 0.9190	0.8500, 0.9170, 0.9680	0.6578, 0.7570, 0.9190
Reliability	0.6570, 0.7650, 0.9050	0.6570, 0.7650, 0.9050	0.4560, 0.5330, 0.7330	0.8500, 0.9170, 0.9680	0.8500, 0.9170, 0.9680
Availability	0.6570, 0.7650, 0.9050	0.6570, 0.7650, 0.9050	0.6578, 0.7570, 0.9190	0.6570, 0.7650, 0.9050	0.4560, 0.5330, 0.7330
Performance	0.6578, 0.7570, 0.9190	0.8500, 0.9170, 0.9680	0.6578, 0.7570, 0.9190	0.6570, 0.7650, 0.9050	0.6578, 0.7570, 0.9190
Availability	0.4560, 0.5330, 0.7330	0.8500, 0.9170, 0.9680	0.8500, 0.9170, 0.9680	0.6570, 0.7650, 0.9050	0.6578, 0.7570, 0.9190
User-Friendly	0.6578, 0.7570, 0.9190	0.6570, 0.7650, 0.9050	0.4560, 0.5330, 0.7330	0.6570, 0.7650, 0.9050	0.4560, 0.5330, 0.7330
Performance	0.6578, 0.7570, 0.9190	0.6570, 0.7650, 0.9050	0.4560, 0.5330, 0.7330	0.8500, 0.9170, 0.9680	0.7720, 0.8560, 0.9450
Operational Cost	0.6490, 0.7640, 0.8800	0.6570, 0.7650, 0.9050	0.6578, 0.7570, 0.9190	0.8500, 0.9170, 0.9680	0.6578, 0.7570, 0.9190
Reliability	0.6570, 0.7650, 0.9050	0.6570, 0.7650, 0.9050	0.4560, 0.5330, 0.7330	0.8500, 0.9170, 0.9680	0.8500, 0.9170, 0.9680
User-Friendly	0.6570, 0.7650, 0.9050	0.6570, 0.7650, 0.9050	0.6578, 0.7570, 0.9190	0.6570, 0.7650, 0.9050	0.4560, 0.5330, 0.7330
Operational Cost	0.6578, 0.7570, 0.9190	0.8500, 0.9170, 0.9680	0.6578, 0.7570, 0.9190	0.6570, 0.7650, 0.9050	0.6578, 0.7570, 0.9190

**Table 4:** Weighted normalized decision matrix.

	SAS Visual Analytics	Tableau	QlikView	Bold BI	DOMO BI
Confidentiality	0.0516, 0.0820, 0.0990	0.0516, 0.0990, 0.1220	0.0630, 0.1140, 0.1310	0.0630, 0.0979, 0.1310	0.0230, 0.0430, 0.0550
Integrity	0.0230, 0.0370, 0.0430	0.0230, 0.0370, 0.0430	0.0630, 0.0979, 0.1140	0.0516, 0.0820, 0.0990	0.0516, 0.0820, 0.0990
Reliability	0.0516, 0.0820, 0.0990	0.0516, 0.0990, 0.1220	0.0630, 0.1140, 0.1310	0.0630, 0.0979, 0.1310	0.0630, 0.0979, 0.1310
Availability	0.0230, 0.0370, 0.0430	0.0630, 0.0979, 0.1140	0.0516, 0.0820, 0.0990	0.0516, 0.0820, 0.0990	0.0516, 0.0820, 0.0990
Performance	0.0230, 0.0370, 0.0430	0.0230, 0.0370, 0.0430	0.0230, 0.0370, 0.0430	0.0230, 0.0370, 0.0430	0.0630, 0.0979, 0.1140
Availability	0.0630, 0.0979, 0.1140	0.0630, 0.0979, 0.1140	0.0516, 0.0820, 0.0990	0.0630, 0.0979, 0.1140	0.0516, 0.0820, 0.0990
User-Friendly	0.0516, 0.0990, 0.1220	0.0630, 0.1140, 0.1310	0.0630, 0.0979, 0.1310	0.0630, 0.1140, 0.1310	0.0630, 0.0979, 0.1310
Performance	0.0230, 0.0370, 0.0430	0.0630, 0.0979, 0.1140	0.0516, 0.0820, 0.0990	0.0630, 0.0979, 0.1140	0.0516, 0.0820, 0.0990
Operational Cost	0.0516, 0.0990, 0.1220	0.0630, 0.1140, 0.1310	0.0630, 0.0979, 0.1310	0.0630, 0.1140, 0.1310	0.0630, 0.0979, 0.1310
Reliability	0.0516, 0.0820, 0.0990	0.0230, 0.0370, 0.0430	0.0230, 0.0370, 0.0430	0.0630, 0.0979, 0.1140	0.0516, 0.0820, 0.0990
User-Friendly	0.0516, 0.0820, 0.0990	0.0516, 0.0990, 0.1220	0.0516, 0.0990, 0.1220	0.0630, 0.1140, 0.1310	0.0630, 0.0979, 0.1310
Operational Cost	0.0230, 0.0370, 0.0430	0.0630, 0.0979, 0.1140	0.0630, 0.0979, 0.1140	0.0630, 0.0979, 0.1140	0.0516, 0.0820, 0.0990

**Table 5:** Separation from positive solution.

	SAS Visual Analytics	Tableau	QlikView	Bold BI	DOMO BI
Confidentiality	0.9050	0.8990	0.9080	0.9360	0.9080
Integrity	0.9360	0.9360	0.9080	0.9280	0.9050
Reliability	0.9050	0.8990	0.9080	0.9280	0.9080
Availability	0.9360	0.9360	0.9080	0.9360	0.9080
Performance	0.9360	0.9080	0.9280	0.9360	0.9080
Availability	0.9280	0.9080	0.8990	0.9080	0.9280
User-Friendly	0.9360	0.9080	0.9360	0.9080	0.9360
Performance	0.8990	0.8990	0.9080	0.9280	0.9360
Operational Cost	0.9360	0.9360	0.9080	0.9280	0.9080
Reliability	0.9080	0.9080	0.9080	0.9360	0.9080
User-Friendly	0.9080	0.9080	0.9080	0.9360	0.9280
Operational Cost	0.9080	0.9080	0.9360	0.9080	0.9360

**Table 6:** Separation from negative solution.

	SAS Visual Analytics	Tableau	QlikView	Bold BI	DOMO BI
Confidentiality	0.0140	0.1420	0.0012	0.1730	0.0150
Integrity	0.1230	0.1420	0.0012	0.0012	0.0012
Reliability	0.0012	0.0150	0.1420	0.1420	0.0012
Availability	0.0140	0.1420	0.0120	0.0150	0.1420
Performance	0.0012	0.1420	0.1420	0.1420	0.0120
Availability	0.1420	0.0012	0.0012	0.1420	0.1230
User-Friendly	0.0150	0.1420	0.0012	0.0012	0.0012
Performance	0.1420	0.0150	0.1420	0.1420	0.0012
Operational Cost	0.1420	0.1420	0.0120	0.0150	0.1420
Reliability	0.0150	0.1420	0.1420	0.0012	0.0012
User-Friendly	0.1420	0.0120	0.0150	0.1420	0.0012
Operational Cost	0.2410	0.1230	0.2410	0.0150	0.1730

**Table 7:** Final ranking of alternatives.

S. No.	Visulization Applications	Closeness Coefficients
1	SAS Visual Analytics	0.52652
2	Tableau	0.53254
3	QlikView	0.48525
4	Bold BI	0.59547
5	DOMO BI	0.57635

**Table 8:** Comparison with Other Methods.

S. No.	Visulization Applications	Method 0 (Proposed)	Method 1	Method 2	Method 3
1	SAS Visual Analytics	0.52652	0.52648	0.52365	0.52456
2	Tableau	0.53254	0.53025	0.53032	0.53789
3	QlikView	0.48525	0.48123	0.48456	0.48321
4	Bold BI	0.59547	0.59946	0.59778	0.59367
5	DOMO BI	0.57635	0.57526	0.57231	0.57149

## Data Availability

On reasonable request, the corresponding author will provide the information supporting the research study’s conclusions.
